# Release of Hepatic Plasmodium yoelii Merozoites into the Pulmonary Microvasculature

**DOI:** 10.1371/journal.ppat.0030171

**Published:** 2007-11-09

**Authors:** Kerstin Baer, Christian Klotz, Stefan H. I Kappe, Thomas Schnieder, Ute Frevert

**Affiliations:** 1 Department of Medical Parasitology, New York University School of Medicine, New York, New York, United States of America; 2 Seattle Biomedical Research Institute, Seattle, Washington, United States of America; 3 Department of Parasitology, University of Veterinary Medicine, Hannover, Hannover, Germany; Washington University School of Medicine, United States of America

## Abstract

*Plasmodium* undergoes one round of multiplication in the liver prior to invading erythrocytes and initiating the symptomatic blood phase of the malaria infection. Productive hepatocyte infection by sporozoites leads to the generation of thousands of merozoites capable of erythrocyte invasion. Merozoites are released from infected hepatocytes as merosomes, packets of hundreds of parasites surrounded by host cell membrane. Intravital microscopy of green fluorescent protein–expressing *P. yoelii* parasites showed that the majority of merosomes exit the liver intact, adapt a relatively uniform size of 12–18 μm, and contain 100–200 merozoites. Merosomes survived the subsequent passage through the right heart undamaged and accumulated in the lungs. Merosomes were absent from blood harvested from the left ventricle and from tail vein blood, indicating that the lungs effectively cleared the blood from all large parasite aggregates. Accordingly, merosomes were not detectable in major organs such as brain, kidney, and spleen. The failure of annexin V to label merosomes collected from hepatic effluent indicates that phosphatidylserine is not exposed on the surface of the merosome membrane suggesting the infected hepatocyte did not undergo apoptosis prior to merosome release. Merosomal merozoites continued to express green fluorescent protein and did not incorporate propidium iodide or YO-PRO-1 indicating parasite viability and an intact merosome membrane. Evidence of merosomal merozoite infectivity was provided by hepatic effluent containing merosomes being significantly more infective than blood with an identical low-level parasitemia. Ex vivo analysis showed that merosomes eventually disintegrate inside pulmonary capillaries, thus liberating merozoites into the bloodstream. We conclude that merosome packaging protects hepatic merozoites from phagocytic attack by sinusoidal Kupffer cells, and that release into the lung microvasculature enhances the chance of successful erythrocyte invasion. We believe this previously unknown part of the plasmodial life cycle ensures an effective transition from the liver to the blood phase of the malaria infection.

## Author Summary

The malaria parasite *Plasmodium* undergoes one large round of multiplication in the liver before beginning the blood phase of the life cycle, the phase that causes the typical episodes of fever and chills. Using intravital microscopy and fluorescent parasites, we studied the mode and dynamics of parasite release from the liver, a critical stage in the malaria life cycle. Earlier work had indicated that infected liver cells could release packets of dozens to hundreds of parasites enveloped by host cell membrane, structures now known as merosomes. We report here that this is the predominant mechanism of parasite release from the liver. The host-derived merosome membrane lacks a marker for phagocytic engulfment, thus allowing safe passage through the gauntlet of Kupffer cells, highly active liver macrophages. Merosomes remain intact during passage through the heart and become sequestered within lung capillaries where the membrane eventually disintegrates liberating the parasites into the lung circulation. We propose that this previously unknown part of the life cycle of *Plasmodium* facilitates red blood cell invasion, thus jump-starting the blood phase of the life cycle and the onset of clinical malaria.

## Introduction

Two billion people, more than one third of the world's population, live at risk for malaria and about 1 billion are infected. Each year there are 300 million to 500 million new cases with 2–3 million deaths, the vast majority young children in Africa. We are now forty years past the discovery that radiation-attenuated sporozoites protect against malaria [1], but we still lack an efficient malaria vaccine to combat this deadly parasitic disease, and drug resistance is wide-spread [2].

The malaria infection begins with the introduction of sporozoites from the bite of an infected *Anopheles* mosquito [3,4]. The sporozoites travel to the liver and develop in hepatocytes to large exoerythrocytic forms (EEFs) [5,6]. Schizogonic division of the EEF then results in the formation of thousands of first-generation merozoites, which are responsible for the initiation of clinical malaria. Merozoites have a short life span and must infect erythrocytes immediately after release into the bloodstream [7]. Merozoites are also highly susceptible to phagocytosis and must therefore avoid contact with macrophages [8]. Acute danger of phagocytic elimination is presented in the form of Kupffer cells [8], the resident phagocytes of the liver that comprise by far the largest population of tissue macrophages of the body [9]. Kupffer cells are predominantly located at sinusoidal bifurcations, largely within and often spanning the sinusoidal lumen [9–11], thereby presenting significant obstacles for non-self particulate material. This strategic position of Kupffer cells makes it difficult for free merozoites to exit the liver without being trapped by these surveillance cells of the innate immune system.

The first evidence suggesting that merozoites can be released from hepatocytes as clusters, held together by host cell cytoplasm, was presented several decades ago in Garnham's ultrastructural examination of Plasmodium yoelii–infected murine livers and described in more detail in Meis' extensive electron microscopic studies on P. berghei infection of the mouse [5,12,13]. More recently, we and others reported that merozoites are released as “extrusomes” or “merosomes” that contain hundreds to thousands of parasites [14,15] (reviewed in [16,17]). Our initial intravital observations using green fluorescent P. yoelii and BALB/c mice revealed extensive movement within EEFs nearing completion of merozoite maturation culminating in budding and release of merosomes into the hepatic bloodstream [14]. An elegant series of in vitro studies described the differentiation of *P. berghei* merozoites in the human hepatoma cell line HepG2 [15]. While developing into hepatic schizonts, the intracellular parasites prevent the initiation of a death program in their host cells, but leave them to die once merozoite formation is complete. Underlying molecular details remain to be determined, but the data suggest that host cell death in this in vitro model shares more features with autophagy than apoptosis or necrosis [18]. However, information on the viability of hepatocytes releasing merozoites into the sinusoidal blood is lacking to date.

Because P. yoelii infection of the mouse represents an accepted model closely reflecting human malaria [19], we used a variety of microscopic techniques to study the dynamics of merosome budding from infected hepatocytes and the fate of hepatic merozoites in the body. Confocal images provided measurements of merosome volume, merosomal merozoite content, and EEF volume, and appropriate mathematic processing of these data allowed us to calculate the number of hepatic merozoites produced by P. yoelii sporozoites in the murine host. Using intravital and ex vivo microscopy, we found that the vast majority of hepatic P. yoelii merozoites leave the liver camouflaged as merosomes, disseminate within the cardiovascular system, and arrest in the lungs. Molecular markers revealed that merosomal merozoites remain viable and infectious until being released into the pulmonary microcirculation. In contrast, various in vivo and ex vivo assays suggest that unreleased merozoites and the exhausted host cell eventually succumb to necrosis. The resulting inflammatory stimulus attracts neutrophils, and mononuclear phagocytes thus give rise to the formation of microgranulomata. Overall, this systematic temporal and quantitative analysis indicates that merosome formation and release by host hepatocytes, merosome transport to and sequestration in the lungs, and release of merozoites into the pulmonary microvasculature are parts of a previously unrecognized phase of the *Plasmodium* life cycle.

## Results

### Morphology of Late P. yoelii Liver Stages

**Figure 1:**
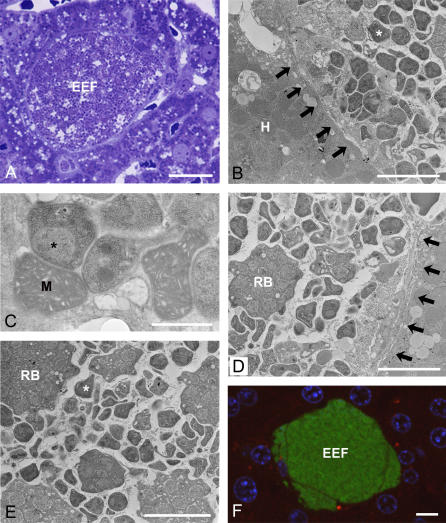
Characterization of Late P. yoelii EEFs (A) Semithin Epon section showing a mature EEF containing thousands of merozoites at 52 h after P. yoelii sporozoite infection. Note that few sinusoids are visible surrounding the EEF. Bar = 10 μm. (B) An infected hepatocyte containing a mixture of merozoites and host cell organelles indicating that the PVM has ruptured. The membrane of the infected cell is in close contact (arrows) with a neighboring hepatocyte. H, hepatocyte. *, merozoites. Bar = 5 μm. (C) Merozoites lie in close apposition to ultrastructurally well-preserved host cell mitochondria. M, mitochondria; *, merozoites. Bar = 1 μm. (D) EEF containing numerous merozoites and a few host cell organelles and remnant bodies. Note that one adjacent sinusoid has collapsed (arrows), while another contains an erythrocyte in its lumen indicating preserved blood flow. RB, remnant bodies. Bar = 5 μm. (E) Merozoites and remnant bodies of various shapes and sizes are embedded in a loose matrix. RB, remnant bodies; *, merozoites. Bar = 5 μm. (F) Intravital micrograph showing a mature PyGFP EEF at the onset of merosome formation. The nuclei of the host cell and the neighboring hepatocytes are visualized by Hoechst staining. Bar = 10 μm.

P. yoelii–infected mice have been suggested to represent a suitable model for human malaria [20]. We also consider *P. yoelii* an appropriate rodent model for liver stage analysis because it induces less inflammation in murine livers than *P. berghei* and produces more EEFs [21]*,* which in addition are generally larger and contain more merozoites [12,22,23] (Table S1). While available for other species such as P. berghei, information is scarce regarding ultrastructural changes during P. yoelii EEF maturation in the liver and the subsequent release of first generation merozoites [12,24]. To help fill this gap and to expand our previous investigation of *Plasmodium* merosomes in live mice [14], we used several light and electron microscopy techniques to examine this process. Mature *Plasmodium* EEFs contained thousands of merozoites enclosed in a parasitophorous vacuole (PV). Up to the final developmental stage and onset of merozoite release, infected hepatocytes remained in close contact with neighboring uninfected parenchymal (Figure 1A and 1B) and sinusoidal cells (Figure 1C). Shortly before merosome formation, the PV membrane (PVM) disintegrated so that host cytoplasm contained a mixture of mature merozoites, morphologically intact hepatocyte organelles (Figure 1D), parasite remnant bodies (or pseudocytomeres [5]), and parasite stroma left over from schizogonic merozoite formation (Figure 1E). Some of the sinusoids adjacent to infected hepatocytes remained filled with erythrocytes indicating preservation of function, but others were compressed by the expanding parasite and lacked erythrocytes suggesting local obstruction of blood flow (Figure 1C). To calculate the merozoite content of mature EEFs (see below), we needed accurate measurements of the EEF size. Compared to tissue sections, intravital microscopy of green fluorescent protein (GFP) Plasmodium yoelii parasite (PyGFP)–infected mouse livers (Figure 1F) offered the advantage of examining live tissue within an intact animal, thus avoiding artifacts associated with both fresh and fixed sections. Mature EEFs within the liver typically have a slightly ellipsoid shape with the minimum and maximum diameters ranging from 40 to 75 μm (with averages of 49.2 ± 10.3 μm to 55.6 ± 9.0 μm), respectively (*n* = 16).

### Mechanism of Exoerythrocytic Merozoite Release

**Figure 2:**
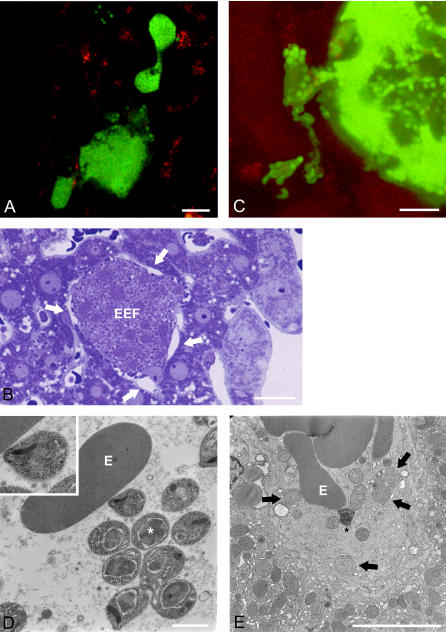
Merozoite Release by Merosome Formation (A) Intravital microscopy of merosomes budding from an EEF. Note that a few individual merozoites are located in the vicinity of the EEF (see Videos S1–S3). Bar = 10 μm. (B) Semithin Epon section showing an EEF that has lost contact with the surrounding parenchyma (arrows). Bar = 10 μm. (C) Projection of an EEF in the process of disintegration; note that the cell releases individual merozoites into the environment (see Video S4). Bar = 10 μm. (D) Electron micrograph showing merozoites located free in a liver sinusoid. The apical pole of one of the merozoites is in close contact with an erythrocyte (insert). E, erythrocyte; *, merozoites. Bar = 1 μm. (E) Liver sinusoid containing free hepatocyte mitochondria (arrows) and a free merozoite. E, erythrocyte; *, merozoite. Bar = 5 μm.

Detailed intravital examination of 30 mice at times ranging from 30 to 74 h after intravenous infection with PyGFP sporozoites allowed us to follow the complex series of events involved in merozoite liberation from hepatocytes. We monitored more than 60 EEFs over this period and observed the earliest merosome budding at 46 h (Figure 2A, Videos S1–S3), a time in general agreement with earlier work reporting the first appearance of P. yoelii in the blood at 45.5 h [12]. Of these 60 EEFs, 20 reached maturity during the observation period and released merozoites, while the rest remained immature. The majority (13) of these 20 EEFs released merozoites by merosome formation. Merosome formation continued until 56 h after infection, thus confirming the asynchronous nature of P. yoelii EEF maturation, a common observation in *Plasmodium*-infected livers [5,25]. Because we infected by intravenous sporozoite injection, the well-known slow release of sporozoites from the mosquito bite site [3,26] alone cannot account for the asynchronicity observed here. For individual EEFs, the process of merosome budding and release lasted several hours during which time the host cell gradually decreased in size and separated from neighboring cells (Figure 2B). In addition to fully formed green fluorescent merozoites, released merosomes contained non-fluorescent remnant bodies and host cell organelles, thus providing further evidence that merosome budding occurs after rupture of the PVM. Eventually, the host cell membrane appeared to lose its integrity and allowed some leftover merozoites to enter the bloodstream singly and without protection by a merosomal membrane (Figure 2C and 2D, Video S4). GFP radiated out from the disintegrating EEF into the surrounding tissue, implying that parasite antigens and host cell cytoplasm were set free as well. Indeed, electron microscopic examination showed free mitochondria in the sinusoidal lumen (Figure 2E). Size and shape of these organelles revealed hepatocyte origin. Eventually, inflammatory cells were attracted to the site of the disintegrating EEF. During phagocytic removal of debris from dead merozoites and host cells, neutrophil granulocytes and mononuclear phagocytes transformed the site of the former EEF into a small granuloma (Figure S1), a structure commonly reported at late stages of *Plasmodium* liver infection [5,8,27–31]. Thus, merosome formation in the liver occurs over a period of about 10 h and is followed by disintegration of the host cell and some leftover parasites, clearance of the remains by infiltrating phagocytes, and production of a small granuloma.

**Figure 3:**
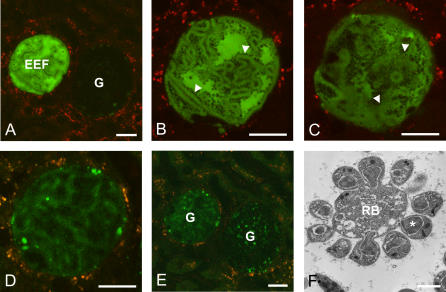
Rapid EEF Decay (A–D) Individual frames from an intravital video showing the process of liver stage transformation into an EEF ghost (see Video S5). (A) The still image shows an intact EEF adjacent to a ghost. G, ghost. (B) Individual frame showing rapid decay of the EEF shown on the left of (A). Note that loss of cytosolic GFP reveals the presence and arrangement of the merozoites (arrow). (C–D) Only a few of the thousands of merozoites remain visible in the faintly fluorescent EEF ghost. (E) Eventually, the infected hepatocyte has transformed into an EEF ghost. Note that both ghosts retain close contact with the surrounding tissue (A–E). Bars = 20 μm. (F) Electron micrograph showing a liver sinusoid with merozoites that are incompletely separated from a remnant body. RB, remnant body; G, ghost; *, merozoite. Bar = 1 μm.

Merozoites were also liberated by a less frequent mechanism. Starting earlier than merosome formation (42 h post inoculation), some infected hepatocytes rapidly discharged their content of merozoites and cell organelles by a mechanism appearing to involve rupture of the cell membrane (Figure 3A–3E, Video S5). In some cases, the process was complete in as little as 5 min; in others it lasted as long as 60 min. Of the EEFs rupturing in this manner, 80% harbored mature merozoites, but 20% had a homogeneous cytoplasm; thus, schizogony had not even begun (Figure S2). Occasionally, electron micrographs showed immature merozoites incompletely separated from remnant bodies yet released into the sinusoidal bloodstream (Figure 3F). This apparent rupture-release left large faintly fluorescent EEF ghosts at the site of the former host cell. Because our intravital observations were based on confocal microscopy, we considered the possibility of phototoxicity playing a role in this rupture-release mechanism. However, since EEF ghosts identical to those resulting from observed rupture were detectable at the very beginning of intravital examination, we could reject that possibility. Because the EEFs did not decrease in volume prior to transformation into a ghost, and we did not find erythrocytes associated with these ghosts, we suspect that the remains of the host cell cytoskeleton, the surrounding extracellular matrix, and/or the sinusoidal cell layer resealed the ghost after merozoite release; thus, preventing the formation of hemorrhages. Similar to the end of the merosome release mechanism (see above), EEF ghosts were infiltrated by inflammatory cells that gave rise to small granulomata. When we combine results from intravital microscopy, showing that both mature and immature EEFs undergo this rapid decay, with our electron microscopy data, showing that some of the rupturing EEFs were immature, we conclude that this rapid release process is a result of abortive EEF development that, in the absence of host cell membrane protection, exposes the parasites to Kupffer cell phagocytosis.

**Figure 4:**
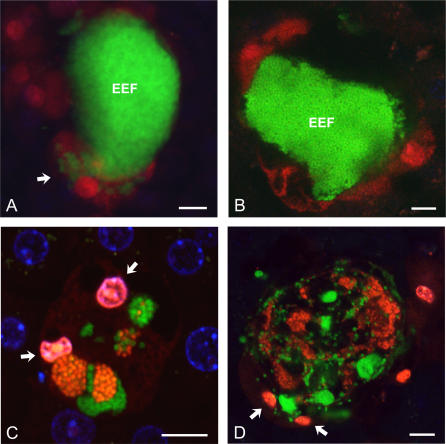
Hepatocyte and Merozoite Viability during Merosome Formation At 52–70 h after infection with PyGFP, mice were intravenously injected with a mixture of the membrane-permeable DNA stain Hoechst 33342 and the dead cell dye PI prior to intravital microscopic examination. (A) Confocal scan showing an intact EEF that has not incorporated PI (red) and is therefore considered viable. Note that merozoites have been discharged into surrounding cells (arrow) some of which have taken up the red dead cell stain. (B) Due to merosome formation, this EEF has decreased in size and detached from the surrounding tissue. The neighboring cells appear to be compressed and are stained with PI (red). (C) A few merozoites are left behind in an EEF that has disintegrated after repeated merosome budding. The two nuclei of the host cell (arrows) have incorporated predominantly PI (red) and some Hoechst stain (blue) and appear pink. In addition, some of the merozoites have lost their green fluorescence and appear red due to PI staining, while others have retained GFP and excluded PI and are therefore viable. The nuclei of the neighboring hepatocytes are visualized by Hoechst staining (blue). (D) EEF at a late stage of disintegration. Some remaining merozoites are viable (green), while other merozoites are dead (red). The nuclei of some surrounding cells have also incorporated PI (arrows). Bars = 10 μm.

To demonstrate that merozoites within merosomes are alive and to help exclude the possibility that merosome release represents an abnormal development, we injected infected mice with markers that reveal cell viability in vivo. At points ranging from 51 to 74 h post inoculation, mice were injected with a mix of the membrane-permeable DNA stain Hoechst 33342 and the dead cell marker propidium iodide (PI). Subsequent intravital confocal microscopy revealed that PI does not enter merosomes or intact EEFs (Figure 4A and 4B), but does stain some of the merozoites left behind in EEF ghosts and also in EEFs that had disintegrated after merosome budding (Figure 4C and 4D). These findings support the interpretations above in that they suggest that merozoites that fail to exit the host cell eventually succumb to necrosis.

Efforts to determine the mode by which merosomes breach the sinusoidal cell layer failed so far due to insufficient numbers of suitable events for analysis. We suspect that budding occurs through the endothelial fenestration rather by a paracellular route, because of the extreme natural variability of the diameter of the fenestrae in response to changes in blood pressure and other physiologic stimuli. Interestingly, mature EEFs were frequently surrounded by a layer of flattened cells that had incorporated the dead cell stain PI (Figure 4A and 4B). Perhaps the death of these cells is due to extreme compression by the extensive expansion of the EEF during the final stage of development. Occasionally, merosomes were found budding into such dead cells, but the immobility of the parasites indicated that they were trapped (Figure 4A).

### Dynamics of Merosome Budding and Transport

**Figure 5:**
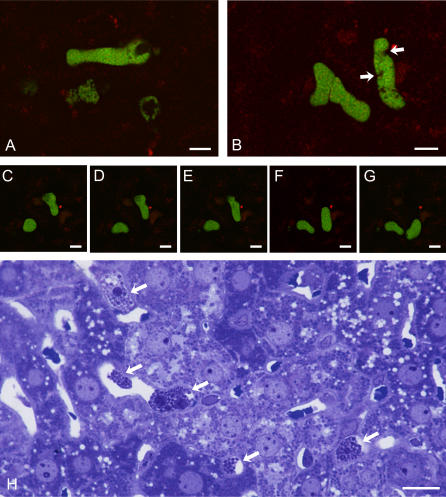
Dynamics of Merosome Transport in the Liver (A) Multiple merosomes separate from an EEF and are rapidly transported towards the central vein (see Video S6). Bar = 10μm. (B) Large merosomes move slowly within the sinusoidal lumen (see Video S7). Remnant bodies can be differentiated from merozoites by their larger size and lack of GFP (arrows). Bar = 10 μm. (C–G) Individual frames of an intravital confocal video showing two smaller merosomes gliding along a sinusoid. Bars = 10 μm. (H) Semithin liver section 52 h after infection with P. yoelii. Multiple merosomes, presumably released from a single EEF, can be found inside the sinusoids (arrows). Note the presence of remnant bodies in merosomes. Bar = 10 μm.

When hepatic merosomes initially bud from infected hepatocytes (Figure 5), they are highly variable in size and contain hundreds to thousands of mature merozoites, while merosomes in blood draining from the liver were smaller and more uniform in size. Intravital microscopy showed very large merosomes moving far more slowly than small ones, which leave the liver lobules at a velocity close to that of blood cells (unpublished data). We frequently observed merosomes hindering the free flow of the blood as they moved along a sinusoid (Figure 5C–5G) as well as being hindered by the vascular architecture. The speed of merosome transport at any instant depended on the diameter and local structure of the sinusoid as well as the size of the merosome. We recorded large merosomes being arrested at sinusoidal bifurcations where they occasionally even reversed direction of movement (Figure 5A and 5B and Videos S6 and S7). Because morphological measurements taken in vitro are subject to artifact and do not reveal in vivo dynamics, we sought a better understanding of sinusoidal architecture using intravital analysis of uninfected transgenic Tie2-GFP mice that have fluorescent vascular endothelia [32]. We found sinusoidal diameters to range from 3.4 μm to 14.1 μm (6.7 ± 1.9 μm; *n* = 94) under normal blood pressure conditions. Although large merosomes greatly exceed this size range, their considerable deformability allowed them to gradually wind their way towards the central vein and exit the liver without rupture and release of merozoites, a process aided by resizing (Figure 5A and 5B). We occasionally observed large merosomes subdividing into smaller ones while traveling through sinusoids (Videos S8 and S9), but we suspect that shear forces associated with the faster blood velocity in larger vessels caused merosomes in the hepatic effluent and inside lung capillaries to be generally smaller and uniform in size compared to those in the liver. The importance of mechanical forces for resizing is demonstrated by another set of experiments in which PyGFP-infected mouse livers were removed from the animals and analyzed ex vivo by confocal microscopy, i.e., in the absence of blood flow. The sinusoids of such livers contained merosomes of an unusually large size (Figure S3A). When livers were perfused with medium prior to ex vivo confocal microscopy, the sinusoids contained even larger merosomes (Figure S3B). We contend that lack of blood flow prevents subdivision of large merosomes into smaller ones and that liver perfusion hastened merosome budding and liberation from the host cell.

**Figure 6:**
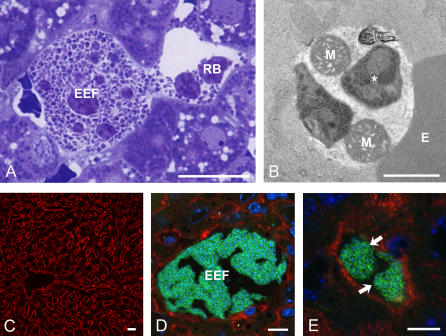
Merosomes Contain Viable Merozoites and Host Cell Organelles (A) Semithin section showing an EEF releasing a merosome into a sinusoid. Note that remnant bodies are a normal component of the merosomal cytoplasm. RB, remnant bodies. Bar = 10 μm. (B) Electron microscopy documents that merosomes contain well-preserved merozoites and hepatocyte mitochondria. E, erythrocyte; M, hepatocyte mitochondria; *, merozoite. Bar = 1 μm. (C) Immunolabeling of frozen liver sections for ASGR-1 reveals the presence of the receptor on the basolateral portion of the membrane of all hepatocytes (red). Bar = 20 μm. Neither EEFs (D) nor merosomes (E, arrows) express ASGR-1 on their surface. The merozoites were visualized with Hoechst (blue) and an antibody against MSP-1 in combination with PA-FITC (green). Bars = 10 μm.

Merosome formation results in packaging a mixture of parasites, remnant bodies, and host cell cytoplasm within host cell membrane for release into the sinusoidal lumen (Figures 5H and 6A). Ultrastructurally, the merosomal matrix contained well-preserved merozoites and morphologically intact host cell mitochondria (Figure 6B) suggesting that these organelles are viable at the time of merosome budding. Merosomes also typically contained remnant bodies (Figure 6A) suggesting that these leftovers from EEF schizogony represent a natural component of the merosomal cytoplasm. In the absence of better viability markers, we interpret the presence of MSP-1 on the surface of merozoites in both mature EEFs and merosomes (Figure 6D and 6E) to indicate intactness and complete differentiation of the parasites, and propose that merosomes are linked to productive infection of erythrocytes. Disintegration of the PVM prior to merosome formation indicates the merosome membrane is derived from hepatocyte cell membrane. Asialoglycoprotein receptor 1 (ASGR1), a protein expressed only on parenchymal liver cells [33–37], was detectable by immunofluorescence lining the basal hepatocyte surface within the space of Disse (Figure 6C). ASGR1 clearly surrounded mature EEFs (Figure 6D), but it was absent from the merosomal membrane (Figure 6E). The lack of this hepatocyte surface protein could be due to dedifferentiation of the infected host cell or modification by the intracellular parasite at late stages of EEF maturation. However, because the ASGR1 label was located predominantly in the space of Disse rather than on the hepatocyte membrane, more work is needed to define the composition of the merosome membrane.

### Merosomes Exit the Liver Intact

Because intravital observations showed that merosomes remain intact during transport towards the central vein, we examined the hepatic venous effluent for membrane-enveloped parasites. To do this, we opened the inferior Vena cava at its point of entry into the diaphragm and collected blood from the peritoneal cavity. Thick smears were prepared from 5 μl blood, and the concentrations of venous merosomes from three separate experiments were measured. In hepatic venous blood collected 52 h post infection with 2.5 × 10^6^ sporozoites, we found 28.7 ± 4.3 merosomes per μl, and 69% of these merosomes contained between 100 and 200 merozoites (unpublished data).

**Figure 7:**
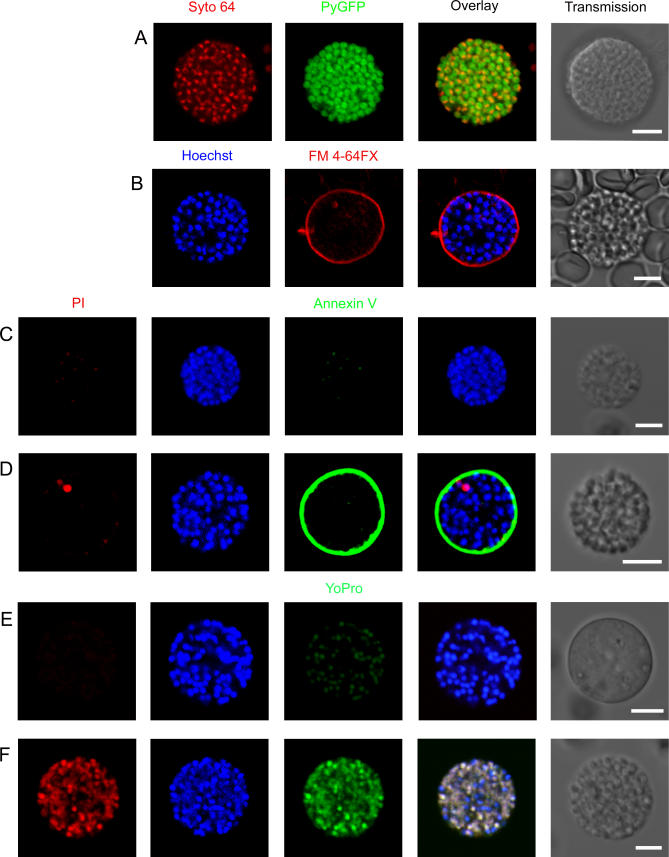
In Vitro Characterization of Hepatic Merosomes (A) Merozoite nuclei in a PyGFP merosome were visualized with the membrane-permeable DNA stain SYTO-64 (red). Differential interference contrast was used to capture the transmission image on the right of the panel. (B) The phospholipid marker FM 4–64FX was used to visualize the membrane surrounding the merosome (red). Merozoite nuclei were stained with Hoechst (blue). (C) Immediately after harvesting from the hepatic effluent, the majority of merosomes were negative for annexin V (green) and PI (red). (D) Prolonged in vitro cultivation led to PS exposure (green) in the outer leaflet of the merosomal membrane and to gradual incorporation of PI (red) into individual merozoite nuclei. Parasite nuclei were visualized with Hoechst (blue). (E and F) Freshly harvested merosomes excluded YO-PRO-1 (green), a DNA stain that selectively passes across apoptotic membranes, and the dead cell stain PI (red). In vitro incubation of the merosomes led to the simultaneous uptake of both nucleic acid stains suggesting that merozoite death occurred by necrosis rather than programmed cell death. Note that colocalization of YO-PRO-1, PI, and Hoechst causes nuclei to appear white. Bars = 5 μm.

While much information is available on P. berghei–infected HepG2 cells [15], in vivo data on the molecular composition of the merosome membrane, for example phosphatidylserine (PS) exposure, are lacking to date. To obtain more detail on merosome structure, another set of experiments was performed in which the parasite material available for examination was enhanced by liver perfusion. Beginning 52 h after infection with PyGFP or wild-type (wt) P. yoelii, livers were perfused with culture medium and the perfusate collected. Cells were immobilized by attachment to Alcian blue–treated glass-bottom dishes and immediately examined by confocal microscopy using conditions that maintain viability. Perfusate merosomes typically adapted a spherical shape in vitro (Figure 7A) and 3-D images from confocal stacks demonstrated a relatively uniform size containing several hundred merozoites. Labeling by the phospholipid marker FM 4–64 FX verified that the parasites were held together by a membrane (Figure 7B).

Immediately after harvesting, the majority of the parasites appeared viable and merosome membranes were negative for annexin V labeling (Figure 7C); thus they do not display PS that targets cells for phagocytosis. However, with increased time in vitro, the presence of PS gradually became apparent (Figure 7D). Merozoites in freshly isolated merosomes did not stain with the dead cell marker PI, but those that became positive for PS also lost the ability to exclude PI (Figure 7D). A further viability assessment utilized YO-PRO-1, a DNA stain that selectively passes through the (intact) plasma membrane of apoptotic cells. Again, merozoites in freshly isolated merosomes did not label with YO-PRO-1, but as time in vitro progressed, they began to incorporate YO-PRO-1 along with PI (Figure 7E and 7F). Within roughly 60 min of in vitro examination, all merosomes were positive for annexin V, YO-PRO-1, and PI. Attempts to quantify the time course of these processes more precisely were prevented by the sensitivity of the merosomes to the various steps of isolation from the liver and concentration by centrifugation. Taken together, these results suggest that P. yoelii merosomes leaving the liver contain viable merozoites, and, similar to P. berghei–infected HepG2 cells [15], lack PS as a membrane marker that signals “eat-me” to phagocytes. Considering that Kupffer cells are located largely within and often spanning the sinusoidal lumen, thus presenting a significant obstacle for non-self particulate material and damaged host cells [9–11], the lack of PS on the merosome membrane is likely critical for merozoite escape from this defense mechanism of the host.

### Merosomes Accumulate in the Lung and Release Merozoites

**Figure 8:**
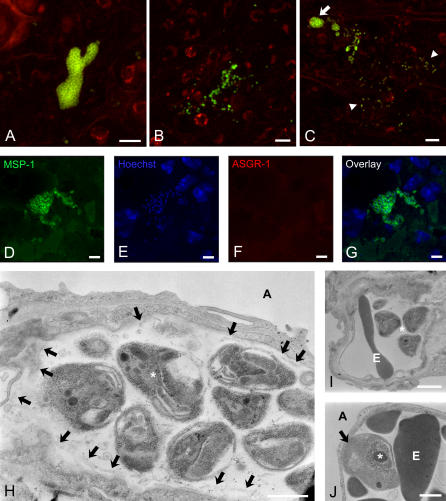
Merosomes Accumulate in the Lungs and Release Merozoites into the Pulmonary Microcirculation (A) Ex vivo confocal microscopy of a mouse lung 52 h after infection with PyGFP shows a large merosome located inside an alveolar capillary. (B) A group of individual merozoites inside pulmonary capillaries. (C) Small parasite aggregates and individual merozoites (arrowheads) fanning out in one direction from a merosome (arrow). This asymmetric parasite distribution suggests that merosomes release merozoites into the pulmonary microcirculation. (A–C) Bars = 10 μm. (D–G) Mouse lung fixed 52 h after infection with P. yoelii. The frozen section was immunolabeled with MSP-1 followed by protein A-FITC (D, green) indicating that the merozoites are intact. Labeling with goat-anti-ASGR-1 followed by rabbit anti-goat IgG-TX (E, red) revealed the absence of the receptor on the merosomal membrane. Nuclei were visualized by Hoechst staining (F, blue). (H) Electron micrograph showing well-preserved merozoites (∗) in the lumen of an alveolar capillary. Only fragments of the merosomal membrane are detectable (arrows). *, merozoite; A, alveolar lumen. Bar = 1 μm. (I) Free merozoites released into the lumen of a pulmonary capillary. *, merozoite; E, erythrocyte. Bar = 1 μm. (J) Alveolar capillary showing an erythrocyte (arrow) harboring a recently invaded merozoite in addition to several uninfected erythrocytes. *, merozoite; E, erythrocyte. Bar = 1 μm.

Because the entire hepatic effluent must pass through the right ventricle and the pulmonary microcirculation before reaching any other capillary bed, we suspected merosomes might sequester in the lungs. To address this, we used ex vivo confocal microscopy to examine the alveolar microvasculature immediately after lung removal while the tissues were intact and the cells alive. At time points from 46 to 58 h after inoculation with PyGFP sporozoites, we found numerous intact merosomes as well as individual parasites (Figure 8A–8C). We did not find pulmonary merosomes earlier than 46 h post inoculation nor later than 65 h, timing consistent with our observation that merosome release begins and ends at roughly 46 h and 56 h. MSP-1 labeling confirmed the maturity of merozoites, both those within merosomes and those already released into pulmonary capillaries (Figure 8D–8G and Video S10). The small liver stage protein UIS-4, which localizes to the PVM [38], was not detected (unpublished data); thus providing more evidence that the PVM is not involved in merosome formation [5]. As for merosomes in the liver, pulmonary merosomes were negative by immunofluorescence for the hepatocyte receptor ASGR1 (Figure 8F and 8G).

In confocal images we often observed an asymmetric arrangement of individual merozoites in relation to lung merosomes and the pattern suggested some of the merosomes were in the process of disintegrating and releasing merozoites into the pulmonary microvasculature just as blood circulation was stopped by lung removal (Figure 8B). Electron microscopy supports the notion of merozoite release by merosomal membrane degradation. Pulmonary merosomes typically contained morphologically well-preserved merozoites, but the cytoplasmic matrix was swollen, and host cell organelles were clearly degenerating. The membrane of lung merosomes was frequently disrupted or barely detectable (Figure 8H) suggesting that free merozoites found in nearby pulmonary microvasculature had just been released before fixation (Figure 8I). The presence of erythrocytes containing newly invaded merozoites (Figure 8J) supports the notion that blood infection occurred in the lungs.

### Merosomes Do Not Disseminate beyond the Lungs

To determine whether merosomes leaving the liver can pass through the lungs and disseminate throughout the body, we analyzed thick smears of blood collected from the aorta and tail vein for merosomes. We also used intravital microscopy, ex vivo imaging, and immunofluorescence microscopy to examine capillary beds of spleen, kidney, and brain of the same mice. While individual small parasites were occasionally detectable, merosomes were completely absent from aorta and tail vein blood and the microcirculation of these organs (unpublished data). These results demonstrate effective retention of hepatic merosomes in the lungs.

### Merosomal Merozoites Are Viable and Infectious

**Figure 9:**
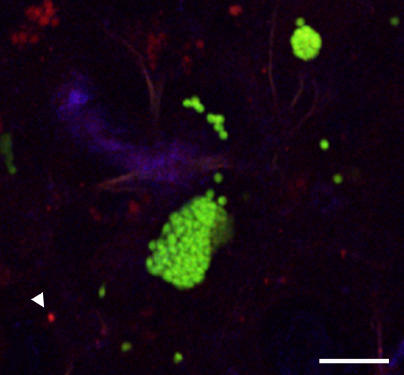
Pulmonary Merosomes Are Viable Mouse lung analyzed ex vivo 48 h after infection with PyGFP. Generally, neither merosomes nor free merozoites incorporated intravenously injected PI indicating that the parasites were viable. Note that one of the merozoites is PI-positive (arrowhead). Nuclei were visualized by Hoechst staining (blue). Bar = 10 μm.

At 52 h after infection with PyGFP, mice were injected with Hoechst 33342 and PI, and the lungs were removed and analyzed ex vivo*.* Confocal microscopy revealed that pulmonary merosomes and free merozoites excluded PI (Figure 9) and were also TUNEL-negative (unpublished data); thus providing evidence of viability. Because infectivity is the ultimate criterion for viability, we tested merosomal merozoites for their ability to induce a parasitemia in naïve mice. However, interpretation of results from inoculation with blood containing merosomes is complicated by the presence of infected erythrocytes. To circumvent this, we initially attempted to eliminate parasitized erythrocytes using selective hypotonic lysis, but this also affected the integrity of the merosomes. Our solution was to control for infected erythrocytes by comparing the infectivity of two types of blood taken from the same mouse: hepatic effluent (with merosomes and some infected erythrocytes) and tail vein blood (without merosomes, but with the same number of infected erythrocytes). At 52 h after intravenous infection with 2.5 × 10^6^ wt P. yoelii sporozoites, hepatic effluent and tail vein blood samples were collected for inoculation into other mice. Parasitemia and merosome concentration were determined by analysis of thin and thick blood smears, respectively. Preliminary studies showed that the parasitemia in recipient mice injected with hepatic effluent blood rose significantly faster compared to control mice injected with tail vein blood (unpublished data) suggesting that merosomes exiting the liver are infectious. This conclusion can be confirmed once a method for merosome purification is available.

### Quantitative Analysis of Extrahepatic Merosomes

Because of the large number of parasites and their high packing density, counting merozoites in EEFs is not feasible, although estimates have been published [6,22,23]. We therefore used measurements obtained from merosomes to calculate the number of merozoites contained in mature P. yoelii EEFs. To do this, we first quantified the merozoite content of a subset of smaller merosomes. We found that merosomes with a diameter of 13.4 ± 2.0 μm contained 134.7 ± 51.6 merozoites. Then, we determined the average effective volume merozoites take up inside merosomes. P. berghei merozoites measure 1.0–1.2 × 1.5–1.7 μm [39–41], but because the parasites are embedded in cytoplasm that also contains parasite remnant bodies and host cell organelles, the effective volume the parasites occupy is larger than their actual volume of 0.78–1.23 μm^3^ (based on the ellipsoid volume v = 4/3 π r_1_ r_2_ r_3_). Using a mathematical algorithm for optimal packing of small spheres in a large sphere [42], we found that P. yoelii merozoites have an effective diameter of roughly 2.2 μm and occupy an effective volume of 5.56 μm^3^ in merosomes. Because intravital and electron microscopy showed that the merozoite packing density and the composition of the cytoplasm was basically identical in merosomes compared to mature EEFs (after rupture of the PVM and mixing of parasites and host cell organelles), we then used the sphere packing algorithm to determine the merozoite content of mature P. yoelii EEFs. Based on the measured EEF diameter of 40–75 μm (see above), the effective merozoite diameter of 2.2 μm, and assuming a round EEF shape, we calculated that P. yoelii sporozoites produce 4,200–29,000 hepatic merozoites (Table S1).

## Discussion

**Figure 10:**
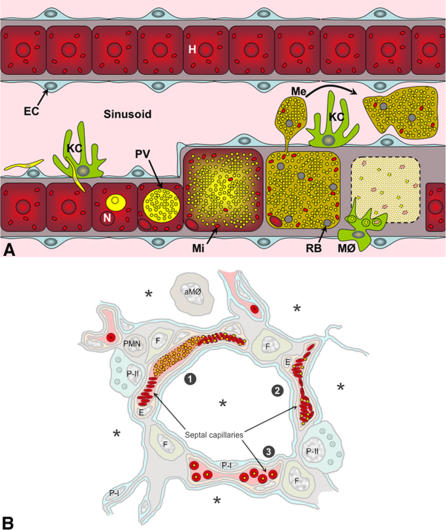
Model of Merosome Dissemination and Merozoite Liberation (A) After malaria sporozoite entry into a hepatocyte, the parasite begins to grow inside a PV to a size larger than its original host cell. Schizogonic division results in the formation of thousands of erythrocyte-infective merozoites. During the final stage of differentiation, the PVM dissolves and allows the parasites to mix with the remaining host cell organelles. Eventually, the plasma membrane of the infected hepatocyte bulges out and forms merosomes; thus releasing merozoites, remnant bodies, and host cell mitochondria into the sinusoidal lumen. Camouflaged by host cell membrane, merosomes are not recognized by Kupffer cells and are shuttled out of the liver. Infiltration of the remains of the infected host cell by mononuclear phagocytes and neutrophil granulocytes gives rise to the formation of a small granuloma. Me, merosomes; RB, remnant bodies; Mi, host cell mitochondria; KC, Kupffer cells; M∅, mononuclear phagocytes; S, sporozoite. (B) Shear forces inside the hepatic and other larger veins cause the merosomes to break down into smaller units. After passing through the right heart, these small merosomes are arrested inside lung capillaries where (1) they eventually release infectious merozoites into the pulmonary microvasculature; (2) local stagnation of the blood flow due to capillary occlusion by merosomes may result in dense erythrocyte packing; and thus (3) facilitate merozoite infection. ∗, alveolar space; H, hepatocyte; N, nucleus; E, endothelium; P-I, type I pneumocyte; P-II, type II pneumocyte; F, fibroblast; aM∅, alveolar macrophage; PMN, polymorphnuclear macrophage.

We present here a new model for the transition from the liver to the blood phase of the malaria life cycle (Figure 10): large merosomes of various sizes bud from infected hepatocytes, enter the hepatic circulation, exit the liver intact, subdivide into smaller more uniform sizes, but otherwise withstand bloodstream shear forces during passage through the right ventricle, and accumulate in the lungs where the merosomes disintegrate and release merozoites to initiate the erythrocytic phase of the malaria cycle. While EEF of avian and reptilian malaria parasites develop in the reticulo-endothelial or hematopoietic systems [43–45], a major evolutionary change occurred with the mammalian malaria parasites, whose EEF mature in hepatocytes. Perhaps the nutritionally rich and immunologically privileged hepatic environment offers advantages, but it also presents a problem for merozoites released from EEFs into hepatic sinusoids: unless they invade an erythrocyte very quickly they face a gauntlet of highly phagocytic Kupffer cells. The location of most EEFs in the periportal area of the liver lobule [46] means they must travel almost the full length of the sinusoid and pass by a large complement of Kupffer cells before escaping into relative safety outside the liver. As proposed previously by us and others [14,15], our premise is that evolution produced a countermeasure to this threat: release of merozoites within large packets that are initially hidden from the host's innate immune system by envelopment with a hepatocyte-derived membrane. Here we show that merosomes are delivered to the pulmonary microcirculation where they are released. We propose that release of merozoites into the lung microvasculature rather than into larger blood vessels is advantageous, because the low macrophage density and the reduced blood velocity with reduced shear forces will enhance the ability of merozoites to invade erythrocytes.

Merosome disintegration in the lungs appears to be the predominant mechanism of merozoite liberation into the bloodstream for the following reasons: (1) In confirmation of previous reports on the asynchronous nature of EEF maturation [5,25], we observed P. yoelii merosome formation in the liver from 46 h to 56 h after sporozoite infection. Assuming a 10-h window of merosome release, roughly 3 ml total blood volume in a 40 g mouse, and a 100% rate of sporozoite infection and EEF development, 2.5 million sporozoites would generate 4,167 maturing EEFs per minute, corresponding to 1.4 merosome-releasing EEFs per μl blood. (2) Assuming that extrahepatic merosomes contain on average 150 merozoites, the roughly 29 merosomes we found per μl venous liver blood should have contained 4,350 merozoites. Since P. yoelii EEFs contain 4,200–29,000 merozoites (Table S1), up to 74% of the total number of merozoites released by 1.4 EEFs per min and μl would have been enclosed in merosomes. (3) A large number of merosomes was arrested in alveolar capillaries suggesting that many merosomes withstand the shear forces inside the central cardiovascular system. Together, these data indicate that a major proportion of the merosome population arrives intact in the lungs and then gradually disintegrates, thus liberating merozoites into the microvasculature. Pulmonary merosomes were detectable in the lungs at least up to 58 h after infection, i.e., beyond the period of release from the liver (46–56 h), suggesting that they remained intact for at least many minutes. Similar to hepatic merosomes, which appeared to be infectious and did not stain with annexin V, YO-PRO-1, or PI, pulmonary merozoites were ultrastructurally well preserved, TUNEL-negative, and did not incorporate PI. Together, these data suggest that merosomal merozoites remain viable until their release into the pulmonary microvasculature. Based on the above assumptions, we propose that merozoite liberation in the lungs represents an integral part of the *Plasmodium* life cycle.

Further support for our premise was found in the following observations and suggestions derived from them. The notion that merozoites shuttled out of the liver within merosomes that are protected from phagocytosis by Kupffer cells [8] was confirmed by demonstrating that murine Kupffer cells do not phagocytoze PyGFP merosomes in vitro (unpublished data), in agreement with the finding that P. berghei merosomes are not ingested by a murine macrophage cell line in vitro [15]. Trager and Jensen's finding that *P. falciparum* merozoite invasion is enhanced by lack of flow and dense erythrocyte packing [47,48] supports our hypothesis that merozoites released within capillary beds have a better chance to invade erythrocytes than those released into larger vessels. We can imagine that capillary occlusion by arrested merosomes could be helpful by causing local stagnation of the pulmonary blood flow. We can also speculate that merosome arrest in lung septal capillaries allows *Plasmodium* to exploit the unique microenvironment of the blood-air barrier. Virtually nothing is known about the biology of the first-generation (hepatic) merozoites, but perhaps transient residence in the lungs provides these parasites with time and a suitable microenvironment to gain infectivity for erythrocytes. The well-oxygenated milieu of the terminal airways and the anastomozed nature of the pulmonary microvasculature [49] likely allow local occlusion of septal capillaries by merosomes without causing the necrotic tissue damage associated with infarction of microvessels in other organs.

Many aspects of the process of merosome formation and release we describe are in agreement with earlier work, but others are not. For example, we found that similar to P. berghei–infected HepG2 cells, which detach in toto from the culture vessel after merozoite differentiation is complete [15], merosomes exiting P. yoelii–infected mouse livers contain viable merozoites and initially do not expose PS on their surface. This confirms earlier predictions [14,15] that merozoites are safely shuttled out of the liver disguised as merosomes. The presence of intact mitochondria in mature EEFs indicates that *Plasmodium* liver stages are able to manipulate hepatocytes in a way that useful organelles (such as mitochondria as a source of energy) are preserved, even after merosome budding. Our interpretation, namely that *Plasmodium* controls certain host cell functions to the last minute, differs from the P. berghei HepG2 cell model, in which the parasites induce death and detachment of their host cells followed by merosome budding [15]. Further, the cell membrane of P. yoelii–infected hepatocytes remains in close apposition to that of neighboring parenchymal and endothelial cells until the very end of EEF differentiation, i.e., up to the onset of merosome budding, as reported [5,12,13,50,51]. As merosomes are produced, the host cell gradually decreases in size and loses contact with neighboring cells as reported [15]. We observed that after releasing merosomes over several hours, the exhausted host cell eventually disintegrates. Some free merozoites still escaped and entered the sinusoidal lumen, thus being exposed to attack by Kupffer cells. In contrast, others proposed that the remaining host cell remnant is rapidly expelled in toto from the tissue with the resulting void immediately filled by neighboring cells [15,18]. We find that the necrotic remnant attracts neutrophils and mononuclear phagocytes, which eventually produce a small granuloma. Such granulomata are a frequent observation in P. yoelii– and particularly in P. berghei–infected mouse livers [5,8,27–31]. Rather than the void created by expulsion of an EEF being filled quickly, our in vivo observations suggest that hours, if not days, are required for phagocytic removal of parasite and host cell debris with subsequent repair of the structural damage before normal tissue architecture is restored.

Although we found merosome formation to be the predominant mode of merozoite release from the liver, we observed a less frequent but still common alternative: EEFs undergoing what we interpret as decay. This alternative process of EEF ghost formation was rapid and typically complete within minutes to an hour. In contrast to merozoite release by merosome formation, ghost-forming EEFs did not detach from the surrounding tissue. EEF decay was accompanied by leakage of GFP into the surrounding tissue suggesting damage to the host cell membrane. It occurred in immature EEFs (recognizable by a homogeneous green fluorescent cytoplasm) and also in mature EEFs (containing fully formed merozoites) without merosome formation regardless of maturity. Sometimes it was found as early as 42 h after sporozoite infection, hours before merozoite differentiation begins. The end result of this alternative process was the formation of large faintly fluorescent EEF ghosts containing some cellular debris and a few dead merozoites. We interpret this rapid conversion of EEFs to ghosts as abortive liver stage development.

Merozoite content of EEFs has historically been difficult to estimate due to the large number of parasites and their high packing density. Based on measurements of the size and merozoite content of small merosomes combined with size measurements of EEFs and an appropriate mathematical algorithm [42], we were able to calculate the number of merozoites in an EEF (Table S2). Under intravital imaging conditions, mature P. yoelii EEFs measured 40–75 μm and the calculated space effectively occupied by a merozoite is a sphere of 2.2 μm diameter. Using this effective size, we calculated that individual P. yoelii sporozoites produce roughly 4,200–29,000 merozoites per EEF. This number is in general agreement with older estimates of EEF merozoite content [5,12,22,23,52–57] (Table S1). An exception is P. falciparum, which produces considerably larger numbers of hepatic merozoites, most likely because of the small size of the parasites. As far as we know, our analysis of the number of merozoites produced in hepatocytes is the first such analysis based on actual merozoite counts and host cell measurements. Precision is limited by variations in measurements, but basing calculations on direct in vivo measurements enhances accuracy.

Earlier studies conducted by us and others had suggested that merosome budding may precede completion of merozoite differentiation [14,15]. One factor that helped lead to this interpretation is that GFP expressed in the parasite stroma can obscure the parasites in mature EEFs. We now show that prior to merosome formation, the signal of the stromal GFP fluorescence equaled that of the merozoite cytoplasm, thus preventing clear definition of parasites enmeshed in the stroma. At the onset of merosome budding, the stromal GFP emission signal decreased abruptly thus revealing the presence of the already formed fluorescent parasites (Figure 3A–3E and Video 5). Two factors contribute to this reduction in fluorescence of material surrounding the parasites: dilution and loss of cytosolic GFP. Dilution of GFP results from PV disassembly and mixing of fluorescent parasite stroma with non-fluorescent host cytoplasm. Loss of GFP is caused by leakage of the fluorochrome into the environment. In agreement with reports that the hepatocyte membrane becomes permeable at late stages of infection with P. berghei [5], we found that merosome-forming EEFs are typically surrounded by a halo of green fluorescence. Optimization of the imaging conditions allowed us to visualize the parasites inside mature EEFs and revealed that merosomes always contain mature merozoites. Thus, merozoites maturation precedes merosome formation.

Depending on the approach used for measurement, the reported diameters of hepatic and pulmonary capillaries vary greatly. For example, when measured in perfusion-fixed liver tissue, the sinusoidal diameter ranged from 4–6 μm to 9–12 μm [58–60]. A crucially important factor is the pressure applied during perfusion fixation, because the sinusoidal diameter is known to vary with changes in blood pressure [61,62]. To determine the sinusoidal diameter under normal blood pressure conditions, we used live Tie2-GFP mice [32], whose fluorescent endothelia clearly delineate the boundaries of the sinusoidal lumen [31]. In agreement with earlier in vivo microscopic studies, which reported a diameter of 6 μm for portal sinusoids and 7 μm for central sinusoids [58], we found by intravital imaging that liver sinusoids measure 6.7 ± 1.9 μm in diameter. Similar differences between fixed and live specimens were reported for the size of alveolar capillaries. While vascular casts of the lung suggested that alveolar capillaries measure 6.69 ± 1.39 μm in diameter [63], intravital measurements determined a functional diameter of only 1–4 μm [64,65]. Regardless which liver sinusoid and lung capillary measurements are relied upon and regardless of the drastic reduction in merosome size after leaving the liver, merosomes still exceed the size of the lumen of the microvasculature of both liver and lung. Since even the largest merosomes were eventually transported out of the liver, the much smaller extrahepatic merosomes would be expected to be malleable enough to be able to pass though the pulmonary capillary bed. Therefore it is somewhat surprising that the lungs effectively clear the blood of all merosomes, so virtually none were detectable in arterial blood harvested from the left ventricle, in the capillary beds of spleen, brain and kidney, or in tail vein blood. The fact that the velocity in pulmonary capillaries is somewhat higher than hepatic sinusoids [66–69] makes this more unexpected. Consequently, the possibility of a receptor-mediated mechanism for pulmonary merosome arrest cannot be excluded.

## Materials and Methods

### Parasites.

Anopheles stephensi mosquitoes were used to propagate wild-type P. yoelii (strain 17 XNL) or PyGFP [14,70]. Sporozoites were purified from the salivary glands of female A. stephensi mosquitoes [71].

### Animals.

Mice were (1) Balb/c (Taconic Farms, Incorporated), (2) Swiss Webster (Taconic Farms, Incorporated), or (3) Tie2-GFP mice, a transgenic strain that expresses GFP in vascular endothelial cells under control of the Tie2 promoter (STOCK Tg(TIE2GFP)287Sato/J; Jackson Laboratory) [31,32]. Animals were maintained and used in accordance with recommendations in the guide for the Care and Use of Laboratory Animals.

### Surgery for intravital microscopy.

Mice were inoculated into the tail vein with 0.3–1.5 × 10^6^ PyGFP sporozoites. At 30–66 h p.i., the animals were surgically prepared for intravital imaging of liver and spleen as described [31] and anesthetized by intraperitoneal injection of a cocktail of 50 mg/kg ketamine (Ketaset, Fort Dodge Animal Health), 10 mg/kg xylazine (Rompun, Bayer), and 1.7 mg/kg acepromazine (Boehringer Ingelheim Vetmedica). Reinjection of the anesthetics at 30-min intervals allowed intravital microscopic examination of the animals for at least 3 h [31].

### Intravital confocal microscopy.

After surgical preparation for intravital imaging, mice were placed onto the stage of an inverted Zeiss DMIRE2 microscope, equipped with a temperature-controlled Ludin chamber, and analyzed with a Leica TCS SP2 AOBS confocal microscope. Appropriate laser lines were used to excite GFP, various other fluorochromes, and the natural autofluorescence of the mouse tissues. Laser power was reduced to a minimum to avoid phototoxicity and bleaching. These optimized conditions allowed continuous scanning of live PyGFP for a period of up to 6 h without any apparent effect on viability. To assess parasite and host cell viability, some mice were i.v. injected with 1–2 μg/ml of the membrane-permeable nuclear dye Hoechst 33342 prior to confocal microscopy. Other mice received 1 μg/ml PI in addition to detect dead host cells and/or parasites.

### Infection with P. yoelii.

Mice were intravenously inoculated with 3 ×10^6^ purified wt P. yoelii or 1 × 10^6^ PyGFP salivary gland sporozoites and various organs were removed at 52 h after infection. Tissue slices were snap-frozen in liquid nitrogen or fixed with PBS containing 4% paraformaldehyde for immunofluorescence labeling of cryosections and with PBS containing 4% paraformaldehyde and 1% glutaraldehyde for electron microscopic examination [72,73].

### Ex vivo organ analysis.

At 30–66 h after infection with PyGFP, major organs such as spleen, brain, kidney, or lung were removed, placed into glass-bottom dishes, and kept moist with medium for confocal microscopy analysis.

### Thick blood smears.

Blood was harvested from (1) the terminal hepatic vein, (2) the aorta, or (3) a tail vein. To increase the probability of detection, ten aliquots of 5 μl blood from each of these sites were spread over an area of 1 cm^2^, allowed to dry, and stained with Giemsa without prior fixation. Merosomes were counted and expressed as average number ± STD. In parallel, the number of merozoites per merosome was determined accordingly.

### Analysis of merosome infectivity.

Two days after infection with 1.5 × 10^6^ wt P. yoelii sporozoites, hepatic effluent and tail vein blood was harvested from the same animal and parasitemia and merosome content were determined using thin and thick blood smears, respectively. 20-μl hepatic effluent, containing 1 × 10^5^ infected erythrocytes plus 167 merosomes, or tail vein blood containing the same number of infected erythrocytes but no merosomes, was intravenously inoculated into Swiss Webster mice (three mice per group) and the parasitemia was monitored daily by Giemsa staining.

### Liver perfusion and merosome immobilization.

To improve the recovery of parasite material from the liver, merosomes were dislodged from hepatic sinusoids by perfusing mouse livers via the portal vein with oxygenated medium at 5 ml/min for 10–30 min. The effluent was collected in two fractions: fraction 1 was collected from the Vena cava inferior and contained mainly red blood cells; fraction 2 was collected from the Vena cava superior after ligation of the Vena cava inferior. The cells were washed and allowed to settle onto cover slips or glass-bottom dishes (WillCo Wells) treated with Alcian blue [74] for live cell imaging. Nuclei of merosomes were visualized with the membrane permeable nucleic acid stains Hoechst 33342 (1–2 μg/ml) or SYTO-64. Nuclei of dead parasites were determined with membrane impermeable PI (1 μg/ml). Merosome membranes were stained with 5 μg/ml FM 4–64 FX (Molecular Probes). Annexin V Alexa Fluor 488 conjugate or YO-PRO-1 (0.1 μM) were used to detect evidence of programmed cell death in live merosomes. Tissue sections were stained with a BrdU TUNEL assay kit (Molecular Probes) according to manufacturer's guidelines.

### Merozoite counting.

Alcian blue–immobilized PyGFP merosomes were fixed and labeled with the red nuclear dye SYTO-64. 3-D stacks were scanned by confocal microscopy and the number of merozoite nuclei was counted using a 3-D object count plug-in of ImageJ (NIH freeware). Merozoite number and merosome diameter were then entered into a formula for efficient packing of equal small spheres in a large sphere ( *n* = 0.7405 [1–2D] / D^3^ + 1 / [2D^2^]; D = d_merozoite_ / d_liver stage_ ) [42] to determine the effective diameter/volume merozoites occupy inside merosomes. Based on these calculations and the diameter of PyGFP liver stages measured by intravital microscopy, the merozoite content of P. yoelii liver stages was estimated in relation to size.

### Immunofluorescence microscopy.

Frozen sections of 10-μm thickness were prepared with a Reichert-Jung Frigocut cryostat. Parasites were labeled with a mAb directed against the P. yoelii merozoite surface protein MSP-1, a kind gift from W. Bergman [75]. A rabbit antiserum, which was originally generated against the PVM-associated protein from P. berghei, but exhibits cross-reactivity with P. yoelii UIS4 [38,76], was used to label the PV in *P. yoelii–*infected hepatocytes. Affinity-purified goat IgG against the murine asialoglycoprotein receptor ASGR1 was from R&D Systems. Incubation with the primary antibodies was followed with protein A conjugated to fluorescein isothiocyanate (PA-FITC; Molecular Probes), anti-goat IgG conjugated to Texas Red (GAR-TR; Molecular Probes), or goat anti-rabbit IgG conjugated to Texas Red (GAM-TX; Molecular Probes) in color-matching fluorochrome combinations. In case of a single FITC label, the specimens were counterstained with 0.1% Evans blue in PBS. Immunofluorescence-labeled frozen tissue sections were examined by confocal microscopy.

### Transmission electron microscopy.

Mouse liver or lung tissue was fixed with 1% glutaraldehyde and 4% paraformaldehyde in PBS, post-fixed with 1% osmium tetroxide and 1.5% potassium hexacyanoferrate, stained en bloc with 1% uranyl acetate, dehydrated in ethanol, and embedded in Epon as described [72,73]. Semithin sections were cut with an RMC MT-7 ultramicrotome and photographs were taken with Kodak Ektachrome 160T slide film using a Nikon FX-35DX/UFX-DX camera/exposure system. Thin sections were post-stained with uranyl acetate and lead citrate and viewed with a Zeiss EM 910 electron microscope [73].

### Image processing.

Electron microscopy negatives and Ektachrome slides were scanned with a Hewlett Packard Scanjet 5370C. All digital, electron, or confocal microscopy images were processed using Image-Pro Plus (Media Cybernetics), Adobe Photoshop (Adobe), and AutoDeBlur (AutoQuant Imaging, Incorporated) software.

## Supporting Information

Figure S1 Infiltration of Disintegrating EEFs by Inflammatory Cells Leads to Granuloma Formation (A) Semithin Epon section showing a granuloma (outlined by arrows) in the liver of a mouse 52 h after infection with P. yoelii. Inflammatory cells have infiltrated the remnants of an EEF. The surrounding tissue shows a normal arrangement of hepatocytes and sinusoids. Bar = 10 μm.
(B) Intravital confocal scan of mouse liver showing an EEF in the process of disintegration. Only a small amount of merozoites (green) is left in the remains of the EEF. Inflammatory cells that have infiltrated the remains of the former EEF are visualized by nuclear staining with Hoechst (blue). Bar = 10 μm.
(C) Electron microscopy identifies the inflammatory cells as mononuclear phagocytes and polymorph nuclear granulocytes. Parasite remains can be detected inside phagolysosomes (arrows) of these phagocytes. Mo, mononuclear phagocytes; PMN, polymorph nuclear granulocytes. Bar = 5 μm.
(D) Part of a granuloma showing necrotic remnant bodies and merozoites (∗) embedded in the loose matrix of a degenerating EEF (arrows). It appears that some parasite material has discharged (arrowheads) into the space between the former EEF and a neighboring hepatocyte. RB, remnant body; H, hepatocyte. Bar = 5 μm.
(10.9 MB TIF)

Figure S2 Ghost Formation after EEF Decay Individual frames from an intravital video demonstrating the events involved in rapid EEF decay.
(A and B) At the onset of disintegration, green fluorescent parasite content streams out of an immature EEF into an adjacent sinusoid (arrow).
(C) Eventually, a large faintly fluorescent ghost is left behind. Note that the membrane of the EEF never lost contact with neighboring cells.
(D) Decay of a mature EEF. The sudden loss of GFP from the parasite cytosol reveals the presence of large numbers of merozoites (arrowheads; see Video S9). Bars = 10 μm.
(6.2 MB TIF)

Figure S3 Liver Perfusion Enhances Merosome Formation (A) Ex vivo confocal analysis of a non-perfused mouse liver. The presence of oversized PyGFP merosomes suggests that the absence of blood flow prevented them from subdividing into smaller units. Bar = 20 μm.
(B) Ex vivo confocal analysis of a perfused mouse liver. Merosomes extend over hundreds of micrometers from the EEF into various sinusoids indicating that liver perfusion augmented merosome budding and separation from the host cell. Bar = 100 μm.
(7.2 MB TIF)

Table S1 Size and Merozoite Content of *Plasmodium* EEFs Comparison of the estimated numbers of merozoites produced by various *Plasmodium* species in different hosts with the calculated data obtained from P. yoelii–infected mice presented in this paper.
(62 KB DOC)

Table S2 Merozoite Content of P. yoelii Merosomes and EEFs Merosomes were collected from the hepatic effluent. A subpopulation of small merosomes was used to measure the merosome diameter in relation to the merozoite content. An algorithm for optimal sphere packing (see Materials and Methods) was then used to calculate the effective merozoite diameter in these merosomes. This effective diameter (2.175 μm) was then entered into the same algorithm to determine the number of merozoites contained in the much larger EEFs in relation to their diameter.
(35 KB DOC)

Video S1 Merozoite Release by Merosome Formation Intravital confocal microscopy showing a medium-sized merosome in the process of budding from an EEF (located outside of the optical plane and therefore not shown) at 52 h after intravenous infection with PyGFP sporozoites. The merosome contains hundreds of merozoites and several non-fluorescent remnant bodies. The video was recorded over a total period of 25 min. During this time, the merosome subdivides into two smaller units.
(9.8 MB AVI)

Video S2 Merosome Budding Shows budding of a large merosome from a mature EEF. Note the movement within the EEF during the budding process. A few individual merozoites are located in the vicinity of the infected hepatocyte. Shows intravital confocal microscopy of a mouse liver 48 h after intravenous infection with PyGFP sporozoites. Total recording time: 2 min and 20 s.
(9.8 MB AVI)

Video S3 Merozoite Release by Merosome Budding An EEF releases merozoite-filled merosomes into the bloodstream. Note the ragged surface of the EEF, which is gradually decreasing in size during the budding process. Total recording time: 2 min and 36 s.
(9.4 MB AVI)

Video S4 Individual Merozoites Are Released from a Disintegrating EEF At 54 h after intravenous infection with PyGFP sporozoites, a disintegrating mature EEF can be seen slowly discharging individual merozoites into the bloodstream. Total recording time: 2 min and 36 s.
(9.7 MB AVI)

Video S5 Rapid EEF Decay Leading to Ghost Formation The video shows the initial stage of EEF ghost formation. The mature EEF suddenly leaks GFP into the environment. At the same time, the GFP emission signal in the EEF stroma decreases revealing the presence of parasites. Shows mouse liver 45 h after intravenous infection with PyGFP sporozoites. Total recording time: 6 min and 31 s.
(9.5 MB AVI)

Video S6 Fast Merosome Transport along a Liver Sinusoid Multiple merosomes are transported towards the central vein. The merosomes bud from an EEF, which is located outside of the focal plane and therefore not visible, and contain mature merozoites and non-fluorescent remnant bodies. Several other merosomes remain stationary during the observation period. The video shows intravital confocal microscopy of a mouse liver 52 h after intravenous infection with PyGFP sporozoites. Total recording time: 7 min and 11 s.
(9.7 MB AVI)

Video S7 Slow Transport of Large Merosomes toward the Central Vein Two large merosomes (on the left) are transported along a liver sinusoid, are temporarily arrested at a sinusoidal bifurcation, and reverse direction of movement. The resulting obstruction of the blood flow prevents another merosome (on the right) from entering the same sinusoid. Note the high malleability of the merosomes. The video shows intravital confocal microscopy of a mouse liver 52 h after intravenous sporozoites infection with PyGFP. Total recording time: 5 min and 23 s.
(9.5 MB AVI)

Video S8 Subdivision of a Larger Merosome into Smaller Units While arrested at a sinusoidal bifurcation, a medium-sized merosome releases a smaller unit which is rapidly swept away by the bloodstream. Note the large number of mature merozoites inside the merosome. The video shows intravital confocal microscopy of a mouse liver 52 h after intravenous infection with PyGFP sporozoites. Total recording time: 2 min and 36 s.
(9.4 MB AVI)

Video S9 Newly Released Merosomes Are Large Branched Structures The animated 3-D stack generated from a perfused mouse liver 52 h after infection demonstrates all aspects of merosome release. Ex vivo confocal analysis shows an EEF (center) that has released numerous merosomes of various sizes into adjacent sinusoids. Note that the largest merosomes are branched and contain hundreds of merozoites.
(10.1 MB AVI)

Video S10 Merozoite Release into the Lung Microvasculature Ex vivo confocal microscopy shows a group of individual merozoites being released from a pulmonary merosome (on the left). Note the mobility of the free merozoites. Shows mouse lung 52 h after intravenous infection with PyGFP sporozoites. Total recording time: 1 min and 18 s.
(9.4 MB AVI)

## Abbreviations

ASGR1: asialoglycoprotein receptor 1
EEF: exoerythrocytic form
GFP: green fluorescent protein
PI: propidium iodide
PS: phosphatidyl serine
PV: parasitophorous vacuole
PVM: parasitophorous vacuole membrane
PyGFP: GFP-expressing Plasmodium yoelii parasite
