# Supplementary material for: Release of Hepatic Plasmodium yoelii Merozoites into the Pulmonary Microvasculature
Source: PLoS Pathog. 2007 Nov 9;3(11):e171. doi: 10.1371/journal.ppat.0030171 (PMC2065874; doi:10.1371/journal.ppat.0030171)
Supplement: Supplementary file 1 [file ppat.0030171.st001.doc]

**Table S1.	Size and merozoite content of *Plasmodium*** EEFs.

| **Species** | **Host** | **Merozoite size [µm]** | **Max. diameter of mature EEF [µm]** | **Merozoite number** | **Ref** |
| --- | --- | --- | --- | --- | --- |
| *P. falciparum* | Human | 0.7 | 60 (up to 80) | 30,000 ± 10,000 | [52] |
|  |  | 0.7 | >60 | 40,000 | [54,55] |
| *P. vivax* | Human | 1.2 | 30 - 70 | 10,000 | [52] |
|  |  |  | 45 | 10,000 | [51] |
| *P. ovale* | Human | 1.8 | 70 - 80 | >15,000 | [53] |
|  |  |  | 60 | 15,000 | [51] |
|  |  |  | 70 | 15,000 | [52] |
| *P. malariae* | Human | 2.0 | up to 85 |  | [52] |
|  |  |  | 55 | 15,000 | [51] |
| *P. cynomolgi* | Simian | 1.0 | >40, up to 100 | 10,000 | [52] |
|  |  | 1.13 |  |  | [56] |
| *P. berghei* | Mouse | 1.5 - 1.7 x 1.0 - 1.2 |  |  | [5] |
|  |  |  | 30 - 45 | 1500 - 8000 | [21] |
|  | White mouse, hamster |  | 24 - 27 | 1500 - 2000 | [22] |
|  | Rat |  | 26 | 4000 - 8000 | [22] |
|  | *T. surdaster* |  | 38 - 57 | 10,000 – 18,000 | [22] |
| *P. yoelii* | Mouse | 1.5 | ~35 | 2000 – 20,000 | [12,22] |
|  | Rat |  | 35-37 | 7500 - 8000 | [22] |
|  | Swiss Webster mouse |  | 40-75 | 4200 – 29,000 | this work |

Comparison of the estimated numbers of merozoites produced by various *Plasmodium* species in different hosts with the calculated data obtained from *P. yoelii*-infected mice presented in this paper.
