# Supplementary material for: Release of Hepatic Plasmodium yoelii Merozoites into the Pulmonary Microvasculature
Source: PLoS Pathog. 2007 Nov 9;3(11):e171. doi: 10.1371/journal.ppat.0030171 (PMC2065874; doi:10.1371/journal.ppat.0030171)
Supplement: Supplementary file 2 [file ppat.0030171.st002.doc]

**Table S2.	Merozoite content of *P. yoelii* merosomes and EEFs.**

| **Diameter [µm]** | **Merozoite number** | **Volume [µm^3^]** |
| --- | --- | --- |
| 9 | 36 | 382 |
| 10 | 51 | 524 |
| 11 | 71 | 697 |
| 12 | 95 | 905 |
| 13 | 123 | 1,150 |
| 14 | 157 | 1,437 |
| 15 | 196 | 1,767 |
| 16 | 242 | 2,145 |
| 17 | 294 | 2,572 |
| 18 | 353 | 3,054 |
| 19 | 419 | 3,591 |
| 20 | 493 | 4,189 |
| 30 | 1757 | 14,137 |
| 40 | 4274 | 33,510 |
| 50 | 8478 | 65,450 |
| 60 | 14799 | 113,097 |
| 70 | 23669 | 179,594 |
| 75 | 29196 | 220,893 |
| 80 | 35521 | 268,082 |

Merosomes were collected from the hepatic effluent. A subpopulation of small merosomes was used to measure the merosome diameter in relation to the merozoite content. An algorithm for optimal sphere packing (see Materials and Methods) was then used to calculate the effective merozoite diameter in these merosomes. This effective diameter (2.175 µm) was then entered into the same algorithm to determine the number of merozoites contained in the much larger EEFs in relation to their diameter.
